# Multidrug-Resistant Tuberculosis in Central Asia and Predominant Beijing Lineage, Challenges in Diagnosis, Treatment Barriers, and Infection Control Strategies: An Integrative Review

**DOI:** 10.3390/antibiotics14070673

**Published:** 2025-07-02

**Authors:** Ulan Kozhamkulov, Sholpan Iglikova, Anar Rakisheva, Joseph Almazan

**Affiliations:** 1 Center for Life Sciences, National Laboratory Astana, Nazarbayev University, Astana 010000, Kazakhstan; 2 National Scientific Center of Phthisiopulmonology of the Republic of Kazakhstan, Almaty 050000, Kazakhstan; shiglikova@mail.ru; 3 Department of Phthisiopulmonology, School of General Medicine, Asfendiyarov Kazakh National Medical University, Almaty 050000, Kazakhstan; asr.kz@mail.ru; 4 Department of Medicine, School of Medicine, Nazarbayev University, Astana 010000, Kazakhstan

**Keywords:** tuberculosis, multidrug-resistant TB, Beijing genotype, Central Asia, whole genome sequencing

## Abstract

**Background**: Multidrug-resistant tuberculosis (MDR-TB) remains a significant public health threat in Central Asia, where rising resistance to first-line anti-TB drugs challenges control efforts. As of 2024, the World Health Organization (WHO) reports that over 2.5% of new TB cases and 18% of previously treated cases are resistant to first-line TB drugs worldwide. **Objectives**: This integrative review synthesizes current evidence on MDR-TB in Kazakhstan, Kyrgyzstan, Tajikistan, Turkmenistan, and Uzbekistan, with a focus on infection control, diagnostic advancements, and evolving treatment strategies. **Methods**: A comprehensive literature search was conducted across five electronic databases: PubMed, Scopus, Web of Science, Embase, World Health Organization (WHO) Global Tuberculosis Database, and ClinicalTrials.gov. A total of 29 articles from Central Asian countries met the inclusion criteria. **Results**: Four main themes were identified: “genetic variability and resistance patterns of MDR-TB strains”; “barriers to effective treatment”; “diagnostic tools”, and “infection control strategies”. **Conclusions**: This review underscores the importance of comprehensive, multifactorial approaches in addressing drug-resistant TB in the region. The implementation of early diagnosis and all-oral treatment regimens has improved adherence in recent studies.

## 1. Introduction

Tuberculosis (TB) continues to pose a serious global health challenge, despite being both preventable and treatable [1,2]. According to the WHO Global TB Report 2024 [2], TB affects more than 10 million people globally, with an incidence rate of 134 cases per 100,000 individuals. In Central Asia, TB and MDR-TB continue to be serious public health risks. For instance, in 2023, Kyrgyzstan reported an incidence rate of 112 cases per 100,000; Kazakhstan, 70 per 100,000 and nearly 6900 new TB cases; Tajikistan, 79 per 100,000; Uzbekistan, 57 per 100,000; and Turkmenistan, 49 cases per 100,000 [2]. Four of the five Central Asian countries are included in the global lists of high-burden countries for MDR-TB. These trends highlight the urgent need for targeted strategies to curb TB transmission in the region. Furthermore, MDR-TB poses additional complications, particularly when DR-TB strains develop resistance to the first-line drugs isoniazid (INH) and rifampicin (RIF) [2]. MDR-TB, characterized by resistance to isoniazid and rifampicin, leads to longer, costlier, and less effective treatments [2,3]. The persistence of drug resistance is fueled by delayed diagnosis, poor adherence to therapy, limited diagnostic coverage, and suboptimal healthcare access—especially in rural or socioeconomically disadvantaged areas [3,4,5,6,7]. The misuse of antibiotics and inconsistent treatment regimens further drive acquired resistance [2,6].

Globally, the genetic diversity of *Mycobacterium tuberculosis* strains has been increasingly studied using molecular techniques such as spoligotyping, MIRU-VNTR, RFLP analysis and, more recently, whole-genome sequencing (WGS). These tools allow for classification into seven human-adapted lineages (e.g., Lineage 2—East Asian/Beijing, Lineage 4—Euro-American) and identification of resistance-associated mutations [1,2,7]. The Beijing lineage, in particular, is widely associated with MDR-TB and heightened transmissibility [1,2].

In Central Asia, strain typing efforts have revealed the dominance of the Beijing genotype, especially in Kazakhstan and Uzbekistan [3,4,5,6,7]. Other circulating lineages include the East-African Indian (EAI)/Central Asian (CAS) lineage and the Euro-American lineage. The studies reviewed in this paper employed molecular methods such as WGS, Line Probe Assays (LPA), and GeneXpert to characterize resistance patterns and identify prevalent genotypes [8]. While diagnostic and treatment strategies have improved, gaps in surveillance, infrastructure, and genomic data continue to hinder MDR-TB control in the region [2,9,10,11,12,13,14,15]. Addressing these challenges requires a coordinated regional approach focused on molecular epidemiology, rapid diagnostics, and equitable access to care.

Therefore, an integrative review is warranted to synthesize fragmented findings across regional studies. This review consolidates regional evidence on MDR-TB to highlight the key epidemiological, diagnostic, and treatment challenges. Finally, the review aims to inform targeted policy interventions and guide future research to improve MDR-TB outcomes in the region.

## 2. Results

**Figure 1:**
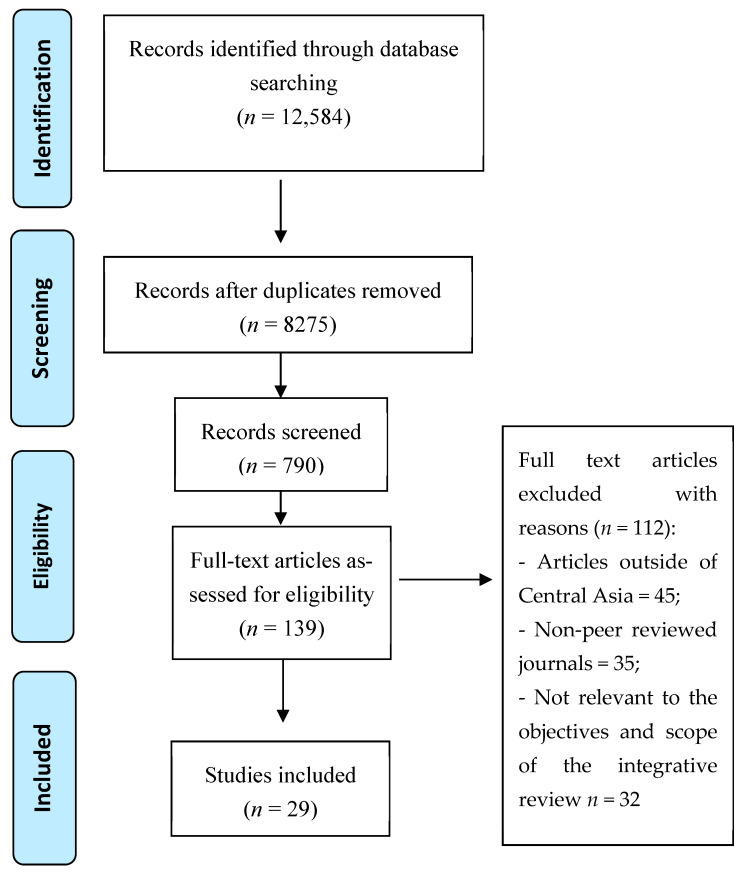
Search flow process adapted from Moher et al. [34].

A total of 29 studies conducted across Kazakhstan, Uzbekistan, Kyrgyzstan, Tajikistan, and Turkmenistan were included in this review (Figure 1). The selected research covers a wide range of topics related to MDR-TB, including genotypic analyses, diagnostic approaches, treatment outcomes, health system challenges, and infection control strategies (see Table 1). The results were categorized into four main themes: Theme 1—genetic variability and resistance patterns of MDR-TB strains; Theme 2—barriers to effective treatment; Theme 3—diagnostic tools; Theme 4—infection control strategies.

**Table 1:** Included studies for the review.

Author/Year/Country	Research Design/Genotype and Resistance	Aims	Data Collection Sampling Population	Analysis	Findings
Akhmetova et al. [4], Kazakhstan	Genotypic study using MIRU-VNTR analysis Cluster Beijing 94-32; *rpoB* (S531L), *katG*, *fabG-inhA*, *oxyR-ahpC* genes of *M. tuberculosis*	To determine the prevalence and genetic characteristics of the Beijing Central Asian/Russian Cluster 94-32 among MDR-TB cases in Kazakhstan	Genotyping of *M. tuberculosis* and MDR-TB strains using mycobacterial interspersed repetitive units–variable number tandem repeats (MIRU-VNTR) analysis *n* = 540 strains New TB cases and MDR-TB patients from multiple regions in Kazakhstan	Genotypic classification of MDR-TB strains, prevalence assessment of Beijing Central Asian/Russian Cluster 94-32	The Beijing Central Asian/Russian Cluster 94-32 was the predominant MDR-TB strain in Kazakhstan, highlighting its role in sustained transmission and drug resistance. The Beijing genotype was associated with drug-resistant TB (*p* < 0.0001), including multidrug-resistant TB (*p* < 0.0001). Among the Beijing isolates, cluster 94-32 showed an association with MDR-TB (*p* = 0.021).
Aye et al. [16], Tajikistan	Descriptive study	To describe the common healthcare-seeking behaviors of new pulmonary TB patients and identify the determinants of delay	Questionnaire and interviews *n* = 204 patients	Principal component analysis and Cox proportional hazards models	The study found that patients who initially sought care from private healthcare providers experienced longer delays in TB treatment than those who accessed the public health system. Private healthcare providers included doctors, pharmacies, and traditional healers. Patients who accessed the public health system, such as district hospitals or health centers, experienced shorter delays in receiving TB treatment. This indicates that the public health system in Tajikistan plays a crucial role in early TB detection and the prompt initiation of treatment. The study identified socioeconomic factors, such as lower education levels and rural residence, as additional barriers to timely TB treatment. These factors contribute to delayed healthcare-seeking behaviors and limited access to healthcare facilities.
Bastard et al. [10], Kyrgyzstan, Uzbekistan (others)	Retrospective multicentric analysis	To analyze and contrast the results of treatment between individuals with HIV-positive and HIV-negative drug-resistant tuberculosis (DR-TB)	Drug susceptibility testing (DST) *n* = 1369 TB patients	Descriptive statistics	The study found that HIV-infected patients with drug-resistant TB had lower treatment success rates, a higher risk of mortality during TB treatment, experienced more frequent and severe adverse events during TB treatment, and experienced longer delays in initiating appropriate treatment compared to HIV-noninfected patients.
Cox et al. [6], Uzbekistan and Turkmenistan	Cross-sectional design Beijing; not specified	To assess the extent of drug resistance in a DOTS program	Survey questionnaire *n* = 213	Univariate and multivariable logistic regression analysis	The data revealed variations in MDR-TB rates between the two regions of Karakalpakstan and Dashoguz. Different proportions of new and previously treated patients were found to have MDR-TB in each region, suggesting potential differences in healthcare access, treatment effectiveness, or disease control measures.
Cox et al. [7], Uzbekistan and Turkmenistan	Cross-sectional design Beijing genotype; non-Beijing strains	To evaluate the prevalence, distribution, and characteristics of the TB Beijing genotype strain	Culture and drug susceptibility testing (DST), IS6110 fingerprinting, and spoligotyping *n* = 397 TB strains	Logistic regression	Fifteen isolates showed mixed banding patterns, indicating the presence of two distinct TB strains in their infections. A total of 382 strains were analyzed, 152 isolates (40%) were grouped in 42 clusters, each consisting of that shared the same fingerprint and spoligotype patterns. The Beijing genotype accounted for approximately half of all isolates. As drug resistance has developed, the prevalence of the Beijing genotype has increased. Of the MDR-TB strains, 75% were genotyped in Beijing, compared to 38% of the completely susceptible isolates.
Cox et al. [17], Uzbekistan and Turkmenistan	Cross-sectional design	To analyzed treatment results of TB patients enrolled in a DOTS program	Sputum smear results *n* = 382 TB patients	Descriptive statistics	In total, 62 of the 382 patients with TB did not respond favorably to treatment. Retesting tests revealed that these patients had the same strain of *M. tuberculosis*, indicating that the therapy had failed. A total of 19 patients had strains that developed new or extra medication resistance. This implies that during treatment, TB strains developed resistance to other medications. In particular, polyresistant Beijing-genotype bacteria exhibited amplified drug resistance.
Cox et al. [18], Uzbekistan	Retrospective observational study	To establish the link between DOTS end-of-treatment outcomes, subsequent TB rediagnosis, mortality, and other factors	Sputum smear-TB positive *n* = 213 patients who were sputum smear-positive	Pearson’s Chi square test, multivariate models	This study revealed a high mortality rate among patients diagnosed with TB. On average, 15% of the patients died per year following diagnosis, with a confidence interval ranging from 11% to 19%. The mortality rate was even higher for cases of MDR-TB, with 43% of MDR-TB patients dying annually. Pansusceptible TB cases have a low mortality rate of 6% per year. Among the new TB cases, 74% were successfully treated. Among the 99 new cases, 25 (34%) were rediagnosed as recurrent TB. Notably, 13 of these individuals were smear-positive upon rediagnosis, indicating an active and potentially infectious form of the disease. The recurrence rate varied depending on the type of TB and the treatment history. Pansusceptible cases had a recurrence rate of 23%, while previously treated MDR-TB cases had a much higher recurrence rate of 60%.
du Cros et al. [19], Tajikistan	Discussion paper systematic framework	To describe the difficulties faced while setting up the TB program and the solutions to these challenges	Case finding within the pediatric hospital and DR-TB facilities	Culture and drug-susceptibility testing analysis	There is a lack of pediatric-specific drug formulations for the treatment of MDR-TB. Healthcare providers often lack sufficient knowledge and training regarding pediatric drug-resistant TB. Central Asian countries may have weak health systems and infrastructure including limited laboratory capacity, inadequate drug supply chains, and poor monitoring and evaluation systems. These factors contribute to the challenges of implementing comprehensive drug-resistant TB programs for children. Recommendations were strengthened for health systems and infrastructure, including laboratory capacity, drug supply chains, and monitoring and evaluation systems.
Daniyarov et al. [11], Kazakhstan	Whole-genome sequencing investigation Beijing; SNPs in resistance-related genes	To assess and describe mutations associated with anti-TB drugs among MDR-TB *M. tuberculosis* clinical isolates	MDR isolates from TB patients, specifically the identification and isolation of pure culture of the pathogen *n* = 8 multidrug-resistant clinical isolates	Whole-genome sequencing and analysis	According to spoligotyping and mycobacterial interspersed repetitive units–variable number tandem repeats (MIRU-VNTR) genotyping, the strains in question are members of the Beijing family. Annotated single-nucleotide polymorphisms, insertions, and deletions of new genomic variations linked to drug resistance have been identified. Genomic variants linked to drug resistance detected.
Darisheva et al. [20], Kazakhstan	Case–control study	To assess the perspective of TB patients household contacts and community dwellers toward ambulatory TB treatment	Index cases based on recently diagnosed pulmonary TB cases (within a 90-day period) *n* = 1083 new pulmonary TB case	Univariate statistics	In total, 24.9% of respondents believed that ambulatory treatment for tuberculosis (TB) was suitable. Favorable views regarding ambulatory TB treatment were connected to factors such as the region where individuals lived, higher educational levels, receiving support from family members, and having previous TB experience. The relationship between TB knowledge and holding a positive attitude towards ambulatory treatment was more pronounced among community controls to TB patients and their family members.
Davis et al. [21], Kazakhstan	Case–control study	To assess the relationship between a history of incarceration, tobacco, alcohol, and drug consumption, and HIV infection and diabetes mellitus with TB	Case–control study *n* = 1600 participant (TB cases = 562, household controls = 515, community controls = 523)	Descriptive statistics a bivariate analysis	Variables such as DM, HIV infection, tobacco use, alcohol use, and incarceration history were associated with TB.
Engström et al. [22], Kyrgyzstan, Tajikistan, and Uzbekistan	Population structure of TB isolates Cluster *M. tuberculosis* Beijing 94-32, 100-32; non-Beijing genotypes	To provide a population overview of *M. tuberculosis* strains’ structure	Solid culture on L-J medium to heat lysis *n* = 607 clinical *M. tuberculosis* (235 from Uzbekistan, 206 from Tajikistan, and 166 from Kyrgyzstan)	Unique multiple 24-loci VNTR analysis (MLVA) MtbC15-9 haplotype	The primary genetic types responsible for the population growth of Beijing strains in Kyrgyzstan, Uzbekistan, and Tajikistan are clusters 94-32 and 100-32, respectively, which play crucial roles in the current MTB epidemic in Central Asia.
Feuerriegel et al. [23], Uzbekistan	Cross-sectional mutations in genes *gyrA*, *gyrB*, *rrs*, and *tlyA* that confer resistance to second-line drugs	To ascertain whether molecular analyses of targeted genes can serve as rapid, specific, and sensitive means of detecting resistance to TB drugs.	All mycobacterial strains from a program for the treatment of MDR-TB *n* = 266 MDR-TB patients (resistant to ofloxacin strains-26 and to capreomycin and/or amikacin-48: Control susceptible to ofloxacin-49 and capreomycin-39)	DNA isolation, PCR, and sequencing	Mutations in *gyrA* or *gyrB* were found in 96% (25/26 strains) of the ofloxacin-resistant strains, while none of the susceptible strains displayed mutations in those two genes. The most frequent mutation in strains resistant to both amikacin and capreomycin was A1401G in *rrs* (34/40 strains (85.0%). Three strains had mutations in *tlyA*, of which two (at codons 18 and 118) were associated with resistance to capreomycin alone. Sequence analysis of short regions within specific target genes is a powerful tool for the rapid detection of resistance to second-line drugs in patients undergoing treatment for MDR-TB.
Hermosilla et al. [24], Kazakhstan	Cross-sectional study	To provide an epidemiological profile of TB among individuals who inject drugs	Baseline interview using questionnaire and biological testing *n* = 728 individuals	Univariate analyses	Older adult males with a history of incarceration and recent drug injection use were more likely to test positive for TB.
Hillemann et al. [25] Kazakhstan,	Surveillance study Beijing and non-Beijing; *rpoB* (RMP), *katG /inhA/ahpC* gene analysis (INH)	To analyze the specific mutations responsible for resistance to rifampicin (RMP) and isoniazid (INH) and strains of MTB	Culture and sensitivity *n* = 142 resistant *M. tuberculosis* strain (92 MDR and 50 INH-resistant, but not RMP-resistant (INHr/RMPs) strains)	Molecular typing, drug-resistance genotyping	The strong similarity of the mutations provides evidence that the transmission of resistant strains plays a significant role in drug-resistance development. A significantly higher proportion of the *rpoB* S531L mutation was found among Beijing genotype strains compared with non-Beijing strains (71.2% vs. 46.2%, *p* = 0.027). In the INHr/RMPs control group, the S315T mutation was significantly more prevalent in the Beijing than in the non-Beijing group (96.9% vs. 71.4%, *p* = 0.012). Strong link between mutations and transmission.
Ibrayeva et al. [26], Kazakhstan	Cross-sectional study Beijing genotype; *rpoB*, *katG*, *fabG-inhA*, and *oxyR-ahpC* genes of *M. tuberculosis*	To assessed the genetic variability of MTB strains and examined their anti-TB drug-resistance profiles	Collection of biological samples, such as sputum or other respiratory specimens *n* = 60 *M. tuberculosis* isolates from prisons *n* = 125 *M. tuberculosis* isolates from the civilian sector	DNA sequencing and analysis, MIRU-VNTR analysis	The percentage of TB strains with unique genotypes collected from civilian patients was 50.4%, whereas among prison patients, it accounted for 31.7%. The discrepancy was found to be statistically significant (χ2 4.42, *p* = 0.035), suggesting a reduced genetic diversity of the TB strain isolates. There was a low genetic diversity of *M. tuberculosis* strains isolated from prison patients compared to civilian patients.
Kaliakbarova et al. [27], Kazakhstan	Descriptive study	To evaluate the impacts of the patient support program on the rates of patient treatment non-adherence	Survey on Psychological and Social Support Provision *n* = 426 MDR-TB patients	Descriptive statistics	Not all TB patients in Kazakhstan have equal access to comprehensive patient support including medical and psychological and counseling, DOT support, social, legal advice, and provision of food packages.
Lalor et al. [13], Uzbekistan	Retrospective cohort	To identify the factors associated with treatment default among patients with multi-drug-resistant (MDR) and extensively drug-resistant (XDR) tuberculosis who started treatment	Patient forms and registers *n* = 710 patients	Univariate analysis, multivariate analysis with logistic regression	The treatment rate increased as the TB treatment program expanded. Patients who had previously interrupted their treatment were more likely to experience adverse outcomes such as death. Health education and high-risk patients’ support, especially after the first 5 months of treatment, may help to reduce treatment default rates.
Merker et al. [15], Uzbekistan	Cross-sectional Beijing; various MDR markers	To study the evolutionary history of *M. tuberculosis* lineage, chronological development of drug resistance, and MDR-TB complex isolates’ transmission networks	Collected *M. tuberculosis* isolates from Karakalpakstan, Uzbekistan *n* = 277 patients	Genome sequencing and Bayesian statistics	The genetic composition of MDR strains poses a significant challenge to the effectiveness of MDR-TB treatments, including the short MDR-TB regimen by the WHO.
Mokrousov et al. [28], Kyrgyzstan	MDR population structure Beijing and non-Beijing genotypes; *rpoB*, *katG*315 and *inhA* promoter region	To evaluate the TB population structure and drug resistance within the civilian population	Culture and sensitivity *n* = 133 adult HIV-negative newly diagnosed pulmonary TB patients *n* = 103 *M. tuberculosis* isolates	Drug-resistance mutation analysis and spoligotyping	The primary families defined by spoligotyping were as follows: Beijing (with 62 isolates), T (with 14 isolates), LAM (with 9 isolates), Ural-2 (with 6 isolates), and Ural-1 (with 3 isolates). Genotypically, 20 isolates exhibited resistance to rifampicin (RIF), 28 displayed resistance to isoniazid (INH), and 17 were identified as having MDR. Then, the drug-resistant isolates were more prevalent in the Beijing group in comparison to the non-Beijing group (*p* = 0.03). Moreover, there was a higher occurrence of the Asia-specific Ural-2 type among individuals in the oldest age group (aged 68 to 85 years; *p* < 0.0001).
Moe et al. [29], Uzbekistan	Retrospective study using programmatic approach	Assess prevalence and risk factors of second-line drug-resistant TB (SLDR-TB) in Karakalpakstan, Uzbekistan	Phenotypic drug susceptibility testing (pDST) data from 2019-2023 *n* = 2405 TB patients who underwent pDST	Multivariable logistic regression models (Allen-Cady approach)	SLDR-TB prevalence: 24%. Risk factors include rifampicin/isoniazid resistance, clofazimine exposure, retreatment status, DR-TB contact, and diabetes
Safaev et al. [30], Uzbekistan	Retrospective observational approach	To record and analyze the trends, attributes, and results of MDR-TB treatment in patients who were enrolled in treatment programs	TB surveillance system that primarily relies on paper records. *n* = 2347 and 2653 MDR-TB patients from 2013 to 2018	Descriptive analysis	The incidence of MDR-TB remained unstable between 2013 and 2018, ranging from 2347 to 2653 cases annually. In contrast, the annual number of extensively drug-resistant tuberculosis (XDR-TB) cases increased sharply from 33 to 433. The annual percentage of MDR-TB patients who successfully completed therapy has declined from 63% to 57% on a nationwide scale. In contrast, the XDR-TB treatment success rate showed a promising upward trend, rising from 24% to 57% annually.
Skiba et al. [14], Kazakhstan	Cross-sectional Beijing 94-32, KAZ-1	To determine the population configuration of the geographic distribution of *Mycobacterium tuberculosis*	Genotyping of *M. tuberculosis* isolates using 24-loci MIRU-VNTR complemented by spoligotyping *n* = 159 clinical isolates of *M. tuberculosis*	Genotyping by using 24-MIRU-VNTR and spoligotyping	The Beijing genotype *M. tuberculosis*, which is associated with MDR, clonal cluster 94-32, and other comparable types, demonstrated a robust MTB population structure, and further research revealed that a recently discovered cluster of viruses known as KAZ-1 may be endemic to the nation. The distribution of KAZ-1 across the nation, with the exception of the south, and the circulation of the NEW-1 family only in the southern region of Kazakhstan suggest a gradient tendency for non-Beijing families. Beijing dominant; KAZ-1 cluster possibly endemic.
Terlikbayeva et al. [31], Kazakhstan	Descriptive study	To identify the significant risk factors of TB including MDR-TB.	Surveillance data from the NTP and the National Institute of Geography (NIG) years 2006–2010	Correlational and descriptive analyses	The study findings revealed contrasting trends between tuberculosis cultures and drug susceptibility testing negatives (CNRs) and MDR-TB cases. Over the study period, there was a decrease in CNRs for tuberculosis, indicating a decline in individuals who tested negative for active TB. In contrast, there was an increase in MDR-TB cases, signifying an increase in tuberculosis strains resistant to multiple drugs. Notably, two specific types of oblasts, Atyrauskaya and Mangystauskaya, displayed significant deviations from the overall trend. These regions experienced substantial decreases in CNRs for TB incidents, indicating a reduction in individuals who tested negative for active TB. Simultaneously, they also observed comparatively large increases in CNRs for MDR-TB incidents, suggesting a notable increase in cases of tuberculosis strains resistant to multiple drugs.
Tilloeva et al. [32], Tajikistan	Cross-sectional study Beijing; not specified	To measure the primary demographic groups within Tajikistan that constituted the new TB cases reported in the year 2017	TB registration data for all new TB case notification *n* = 5182	Descriptive analysis	This study identified several subpopulations among newly reported tuberculosis (TB) cases in Tajikistan in 2017. These subpopulations included migrant workers (728 cases, 70.7%), individuals with diabetes (162 cases, 15.7%), HIV-positive individuals (138 cases, 13.4%), heavy drinkers (74 cases, 7.2%), drug users (50 cases, 4.8%), ex-prisoners (50 cases, 4.8%), and homeless individuals (9 cases, 0.9%). Among these key populations, 307 patients (29.8%) had smear-positive TB, 145 patients (14.1%) had drug-sensitive TB, and 116 patients (11.3%) had MonoDR/MDR-TB. The majority of smear-positive cases (303 patients, 98.7%) initiated treatment within five days.
Ulmasova et al. [5], Uzbekistan	Nationwide survey	To establish the frequency of MDR-TB among TB patients	Survey questionnaire *n* = 1037 patients	Meta-analysis	Several factors were significantly linked to MDR-TB such as being 45 years of age and below (adjusted odds ratio: 2.24; 95% CI: 1.45–3.45), incarceration history (1.93; 95% CI: 1.01–3.70), previous treatment (4.45; 95% CI: 2.66–7.43), and homelessness (1.79; 95% CI: 1.01–3.16).
Usmanova et al. [9], Uzbekistan	Cohort study	To assess the application of treatment regimens for MDR and rifampicin-resistant (RR)-TB	Survey questionnaire *n* = 1481 patients	Log-binomial regression, adjusted risk ratios	Standardized regimen utilization showed a significant increasing trend, from 2% in 2012 to 44% in 2018. Compliance with weight-based drug dosages was observed in 85% of the patients during the intensive phase and 84% during the continuation phase. Approximately 42% of the patients had a prolonged intensive phase. Treatment modifications were made in 44% of the patients during the intensive phase and 34% during the continuation phase. The documentation of treatment document changes was initially suboptimal, ranging from 42% to 75% from 2012 to 2014 but significantly improved in later years, ranging from 86% to 100%.
Van den Hof et al. [33], Kazakhstan (others)	Cross-sectional survey	To measure the financial expenses incurred by patients with MDR-TB	Survey questionnaire and structured interviews *n* = 406 MDR-TB patients	Descriptive analysis	The approximate overall cost for patients with TB amounted to USD 929. The financial burden was further intensified by income reduction, ranging from 38% to 92% of TB patients reporting a decline in earnings and job loss due to the illness.
van Kampen et al. [8], Kazakhstan	Descriptive study	To measure the effectiveness of Xpert as compared to traditional diagnostic techniques in detecting rifampicin-resistant tuberculosis (RR-TB) cases across different risk groups	Smear microscopy, solid media culture *n* = 5611 Xpert MTB/RIF	Descriptive analysis	A total of 5611 Xpert tests were conducted, primarily focusing on who had been in contact with MDR-TB patients, those categorized as “other” presumptive MDR-TB cases, and patients undergoing retreatment (accounting for 26%, 24%, and 22% of the tests, respectively). The Xpert test demonstrated a positive predictive value of 93.1% and 96.4% for detecting rifampicin-resistant TB (RR-TB), while the negative predictive value was 94.6% and 92.7% using solid and liquid culture media.

### 2.1. Theme 1: Genetic Variability and Resistance Patterns of MDR-TB Strains

The genetic diversity of MDR-TB strains *M. tuberculosis* in Central Asia plays a significant role in the development and transmission of resistance (Figure S1). Across Kazakhstan, Uzbekistan, Kyrgyzstan, and Tajikistan, the Beijing genotype of the East Asian lineage (Lineage 2) is the predominant genotype (50–72%) associated with MDR-TB [4,7,14,22,25,26,28,33]. This lineage is associated with high virulence, enhanced transmission efficiency, and increased adaptability under antibiotic pressure. The population structure of non-Beijing genotypes of *M. tuberculosis* belongs to the Euro-American lineage (Lineage 4), with a range of 28 to 45% (LAM, Haarlem, Ural, T, Cameroon, NEW-1, X, S, TUR). The East-African-Indian lineage (Lineage 3) in Central Asia is represented at a low frequency by the Delhi/CAS genotype (0.2–5%) [22,25,26,28].

Cluster analysis of *Mycobacterium tuberculosis* continues to identify two predominant Beijing strain clusters, 94-32 and 100-32, across Central Asia. The 94-32 cluster is consistently represented across all Central Asian countries, with its prevalence ranging from 22.9% to 45.7% [4,14,22]. In Tajikistan, the Beijing 100-32 cluster is more prominent (24.8%) compared to the 94-32 cluster (23.8%) [22]. Conversely, a markedly different distribution is observed in Kazakhstan, Kyrgyzstan, and Uzbekistan, where the 94-32 cluster is found 16, 5, and 4 times more frequently, respectively, than the 100-32 cluster (1.85%, 5.1%, and 4.2%) [4,22]. In Kazakhstan, studies report that up to 88% of MDR-TB strains belong to the Beijing genotype, with 50.3% to 62.5% attributed specifically to the Central Asian/Russian 94-32 cluster; the KAZ-1 sublineage is also increasingly detected [3,4,14,26]. These strains are widely distributed in both community and institutional settings, particularly in correctional facilities, where *M. tuberculosis* strains exhibit low genetic diversity [4,21,26].

In Uzbekistan, Cox et al. [7] and Lalor et al. [13] found that the Beijing strain dominates MDR-TB cases, with 32% of isolates carrying pre-XDR or XDR-related mutations. This highlights a clear genetic shift toward higher levels of resistance, underscoring the need for routine second-line drug susceptibility testing (DST). Kyrgyzstan and Tajikistan also show a high prevalence of Beijing strains, particularly in correctional facilities and among retreatment cases [22,26]. Although Turkmenistan has limited published data, Cox et al. [7] identified Beijing strains as predominant. Thus, the data demonstrate the dominance of resistant TB strains, especially MDR-TB, in Central Asia, with the Beijing lineage (clusters 94-32 and 100-32) playing an important role in the current transmission of MDR-TB in the region.

Resistance is commonly linked to mutations in *rpoB* (e.g., S531L) for rifampicin resistance; *katG* and *inhA* for isoniazid resistance; *gyrA* and *rrs* for second-line drug resistance; and *rpoC* and *rpoA* as compensatory mutations enhancing strain fitness [4,14,15,16,17,18,21,22,23,25,28,32,34]. Molecular typing tools such as MIRU-VNTR, spoligotyping, and whole-genome sequencing (WGS) have been widely applied to characterize regional strain distribution and track transmission (see Table 1). 

Although genotypic insights are essential for understanding resistance mechanisms, the effectiveness of MDR-TB control in Central Asia is still largely determined by systemic barriers that impede diagnosis and timely treatment.

### 2.2. Theme 2: Barriers to Effective Treatment

The management of MDR-TB in Central Asia is impeded by overlapping yet context-specific barriers, including financial hardship, limited infrastructure, diagnostic delays, and social stigma (Table 2). Across the region, access to specialized TB centers remains limited, particularly in rural Uzbekistan and Tajikistan, where diagnostic delays are prolonged due to patients initially seeking care from informal or private providers rather than the public health system [9,10,16]. In contrast, Kazakhstan and Kyrgyzstan, despite being relatively better resourced, continue to face systemic challenges such as drug shortages and healthcare workforce limitations, which disrupt treatment continuity [2,31].

Financial barriers are widely reported across all five countries but are particularly severe in Kazakhstan, where the average out-of-pocket cost per MDR-TB patient exceeds 900 USD. In this context, 92% of patients reported a loss of income or employment during treatment, which directly contributes to poor adherence and treatment dropout [33]. A similar economic vulnerability is observed in Uzbekistan, where the cost of second-line drugs remains a significant reason for treatment discontinuation unless subsidized by donor-supported programs [31]. Health literacy issues and stigma further exacerbate treatment delays. In Tajikistan, misinformation and the fear of high medical costs discourage early care-seeking, especially among rural and migrant populations [10,16]. Studies in Kazakhstan and Uzbekistan report persistent stigma, particularly among incarcerated and marginalized groups, that leads patients to hide their illness or delay diagnosis [16,33].

In terms of diagnostic readiness, countries like Uzbekistan and Kazakhstan have documented a 24% SLDR-TB prevalence among rifampicin-resistant patients, underscoring the need for baseline DST [31]. However, the routine DST capacity remains inconsistent in Tajikistan and Turkmenistan, mainly due to weak laboratory infrastructure and supply chain instability [2,19]. Moreover, psychosocial and nutritional support programs remain underdeveloped across most countries, despite being identified as essential for maintaining treatment adherence. For instance, only a fraction of MDR-TB patients in Kazakhstan reported access to structured counselling or food support [24], and similar service gaps were noted in Uzbekistan [29], Kyrgyzstan and Tajikistan [27].

Given these diagnostic delays, the following section evaluates the effectiveness, accessibility, and limitations of the molecular tools that are central to improving timely MDR-TB detection and treatment outcomes in the region.

### 2.3. Theme 3: Diagnostic Tools

Molecular diagnostics have significantly advanced the detection and management of MDR-TB across Central Asia, yet access and implementation remain uneven (see Table 2). GeneXpert MTB/RIF, a widely used nucleic acid amplification test, enables the simultaneous detection of *Mycobacterium tuberculosis* and rifampicin resistance within hours. Its use has been reported across Kazakhstan, Uzbekistan, and Kyrgyzstan, facilitating early diagnosis in high-burden settings [8,11]. Similarly, Line Probe Assays (LPAs), which detect resistance mutations in *rpoB*, *katG*, and *inhA*, provide results within 24–48 h and are integrated into routine care in several urban facilities [2,25]. WGS has been applied in Kazakhstan and Uzbekistan to track transmission dynamics and resistance profiles with high resolution [11,28]. However, reliance on phenotypic DST persists in rural areas of Kyrgyzstan and Uzbekistan, where limited laboratory infrastructure and staffing constrain molecular diagnostics [9,29].

Emerging tools like next-generation sequencing (NGS) and digital PCR (dPCR) remain underutilized due to high cost, lack of trained personnel, and infrastructure deficits [11]. Supply chain issues—such as GeneXpert cartridge shortages—have disrupted services in Kazakhstan and Tajikistan, thereby prolonging diagnostic timelines and delaying the initiation of appropriate treatment [8,10,31]. Kyrgyzstan has begun implementing targeted next-generation sequencing (tNGS) for drug-resistant tuberculosis, in alignment with the WHO recommendations for the integration into national TB diagnostic algorithms. Several countries, including Indonesia, Namibia, Eswatini, Kyrgyzstan, South Africa, and Mexico, have progressed from pilot initiatives to full-scale implementation as part of their efforts to achieve the End TB Strategy goals by 2030.

While diagnostic innovation is crucial for the timely detection of MDR-TB, effective control also depends on strong infection-prevention strategies, especially in healthcare settings where nosocomial transmission poses a significant threat.

### 2.4. Theme 4: Infection Control Strategies

Infection control is critical to MDR-TB containment across Central Asia, yet implementation varies significantly by country and setting. Hospital-based measures such as N95 respirators, ultraviolet germicidal irradiation (UVGI), HEPA filters, and negative-pressure isolation rooms have been implemented in Kazakhstan and parts of Uzbekistan and Kyrgyzstan, particularly in tertiary TB centers [19,24,27,29]. However, rural facilities often lack these resources, necessitating the development of broader facility-wide protocols, including improved ventilation, early case detection, and biosafety training [6,19]. Kazakhstan has reported strong infection control policy alignment with WHO guidelines, supported by multidisciplinary airborne infection control teams and institutional protocols [24]. In contrast, Tajikistan and Turkmenistan have fewer documented initiatives, and constraints in staffing and infrastructure limit sustained implementation [16].

Diagnostic expansion has also contributed to improved infection control. The introduction of GeneXpert MTB/RIF and digital radiography has enhanced early detection efforts in Kazakhstan and urban areas of Uzbekistan; however, access remains limited in remote regions [19]. Drug susceptibility testing (DST) and the DOTS strategy continue to serve as foundational elements of TB control, particularly in high-burden districts [11,17,19]. Since 2021, the updated WHO guidelines have prompted the regional adoption of all-oral MDR-TB treatment regimens. By 2023, all five Central Asian countries had phased out injectable agents, transitioning to newer drugs such as bedaquiline, delamanid, linezolid, and clofazimine [2,11,12]. Implementation has been the most robust in Kazakhstan and Kyrgyzstan, while Uzbekistan and Tajikistan continue to face challenges related to drug availability and training of the healthcare workforce [2,10,33]. Non-clinical factors also significantly influence treatment outcomes. Community-based adherence support and psychosocial services have been well-documented in Kazakhstan and parts of Uzbekistan; however, such services remain limited in other areas. Legal advocacy, public education, and social protection measures are underutilized, despite their considerable potential to reduce treatment default rates [13,20,24]. Furthermore, cross-country collaboration, standardized infection control protocols, and targeted investments in diagnostics and workforce training are essential for sustainable MDR-TB control across Central Asia [16,30].

This theme underscores the complex interplay between molecular characteristics, systemic barriers, diagnostic capacity, and clinical practices in shaping MDR-TB outcomes in the region.

## 3. Discussion

This integrative review evaluated the genetic diversity of MDR-TB strains, diagnostic limitations, treatment barriers, and infection control strategies in Central Asia. Identifying these determinants is essential for designing targeted interventions and improving MDR-TB control efforts. This section explores critical findings from the reviewed literature, along with systemic gaps and implications for future MDR-TB control.

Several studies from Central Asia report recurring patterns in MDR-TB epidemiology and management. The Beijing genotype, particularly cluster 94-32, emerges as the predominant strain in Kazakhstan, Uzbekistan, Kyrgyzstan, and Tajikistan, strongly linked to high transmission and drug-resistance rates. Additionally, multiple studies reported high rates of treatment failure, recurrence, and mortality among patients with MDR-TB, especially those coinfected with HIV or with histories of previous treatment. Diagnostic delays due to weak laboratory infrastructure and limited access to rapid molecular tools, like Xpert MTB/RIF, were also common. Socioeconomic factors—such as incarceration, substance use, rural residence, and poor healthcare access—frequently contributed to late diagnosis and poor adherence. Together, these findings reveal a region-wide pattern of biological, clinical, and systemic challenges, underscoring the need for coordinated genomic surveillance, targeted healthcare investment, and context-specific intervention strategies.

Several of the included studies acknowledge methodological limitations that compromise the reliability and generalizability of their findings. A key concern is the quality and accuracy of self-reported data, particularly in patient-recall studies. For example, Aye et al. [16] highlighted those patients in Tajikistan often struggled to recall symptom onset and care-seeking timelines, introducing recall bias and inconsistencies. Similarly, retrospective data collection from TB program records may fail to capture seasonal fluctuations in case detection or changes in care accessibility, leading to the further misrepresentation of trends. These findings underscore the need for standardized, prospective, and year-round surveillance protocols to ensure consistent and reliable epidemiological reporting.

In addition to data quality, diagnostic protocol inconsistencies and laboratory reporting errors also pose significant challenges. Cox et al. [7], for instance, reported that negative post-treatment sputum smear results were often misclassified due to the submission of saliva rather than sputum samples. This type of mislabeling can falsely indicate treatment success, leading to inappropriate clinical decisions. These issues point to the urgent need for improved quality control measures, laboratory training, and standardized specimen collection procedures.

Another limitation relates to gaps in genomic and resistance surveillance infrastructure. Daniyarov [11] noted that the lack of access to high-quality clinical isolates and sequencing platforms resulted in small sample sizes, thereby restricting the generalizability of the findings. The absence of robust second-line DST data in some studies further hampers the accurate mapping of resistance patterns [10,20,21,22]. To overcome these limitations, investments in regional sequencing centers, workforce development, and WGS integration into routine MDR-TB care are recommended [11,13,26].

Expanding genomic research capacity and setting up regional sequencing centers could address these limitations, enabling a more robust understanding of MDR-TB evolution in Central Asia [11,15]. Integrating WGS into routine MDR-TB diagnostics has the potential to enhance resistance detection, strain classification, and individualized treatment planning [11,13,28]. By adopting genomic-based approaches, healthcare systems can enable the earlier detection of drug-resistant strains and implement more effective, personalized treatment strategies. Strengthening sequencing infrastructure, workforce training, and accessibility to molecular tools would be essential in advancing MDR-TB management and surveillance efforts in the region.

Beyond methodological limitations, several studies also emphasize contextual and systemic barriers that affect the implementation and outcomes of MDR-TB care. In particular, socioeconomic and geographic constraints significantly delay diagnosis, reduce adherence, and complicate long-term treatment. For example, prolonged therapy disrupts daily life, especially for children, leading to school dropout, emotional stress, and social isolation [19,24,33]. Studies by du Cros et al. [19] and Van den Hof et al. [33] further highlight the indirect costs of illness, including income loss, household strain, and poor reintegration into education or work.

Geographic barriers are another consistent concern, especially in rural and nomadic communities where access to TB services is sparse. Darisheva et al. [20] documented how such gaps result in underreporting and missed diagnoses, while Terlikbayeva et al. [31] identified mismatches between official NTP figures and field-level evidence, suggesting registration and case definition discrepancies. Collectively, these findings underscore the need for expanded geographic coverage, equitable access to healthcare services, and harmonized reporting systems across the region.

In response to the region-wide challenges of tuberculosis control, the World Health Organization (WHO) Regional Office for Europe, in collaboration with Kazakhstan, Kyrgyzstan, Tajikistan, Turkmenistan, and Uzbekistan, launched the TB-Free Central Asia Initiative on 7 April 2025, in Astana [35,36]. This high-level strategic platform aims to eliminate TB, including drug-resistant forms, by 2030. Its core objectives include diagnosing at least 95% of new and relapsed TB cases using WHO-recommended rapid molecular diagnostics, scaling up shorter, all-oral treatment regimens to achieve a treatment success rate of ≥85%, integrating TB care within primary healthcare systems, and preparing for the introduction of new TB vaccines. The WHO European Centre for Primary Health Care in Almaty has been designated to coordinate technical support and oversee implementation efforts [35,36].

Nevertheless, this review provides novel insights into the underexplored role of genomic surveillance in identifying emerging patterns of drug resistance in Central Asia. It further highlights the value of integrating molecular diagnostics with patient-centered care strategies to improve adherence and treatment outcomes [8,11,29]. Additionally, the findings underscore the urgent need to standardize national TB surveillance systems in line with the WHO frameworks to enable more accurate burden estimates and inform targeted, evidence-based interventions. To move beyond current limitations, future MDR-TB control in Central Asia should prioritize the integration of innovative, locally feasible interventions. For instance, deploying mobile health (mHealth) tools including SMS-based adherence reminders, teleconsultations, and digital treatment support platforms may strengthen outpatient care and retention in rural settings. Portable genomic technologies such as nanopore sequencing can support real-time resistance surveillance even in low-resource laboratories. Regional coordination centers for data sharing, combined with investment in community health workers and decentralized care models, could improve early detection, reduce transmission, and sustain long-term treatment adherence. These innovations, adapted to the Central Asian context, present an opportunity to transform fragmented MDR-TB services into a more integrated, data-driven, and equitable system.

## 4. Materials and Methods

This integrative review examined the evidence on MDR-TB genetic variability, diagnostic and treatment challenges, and infection control strategies [37]. This review synthesizes diverse data such as quantitative, qualitative, and mixed-methods studies, allowing for a comprehensive evaluation of MDR-TB management in the region [37]. To ensure methodological rigor, the review followed PRISMA guidelines, and a PRISMA flow diagram (Figure 1) was used to illustrate the literature selection process [34].

### 4.1. Inclusion and Exclusion Criteria

Studies were included if they met all of the following criteria:

- Focused on MDR-TB in at least one Central Asian country;
- Employed empirical research methodologies, including quantitative, qualitative, mixed-methods, randomized controlled trials (RCTs), retrospective cohort studies, or observational studies;
- Peer-reviewed and published in English.

Excluded studies:

- Focused on drug-resistant TB in regions outside of Central Asia;
- Did not present original research data (e.g., reviews, editorials, opinion papers, or discussion-only frameworks);
- Were published as conference abstracts, book chapters, dissertations, or unpublished theses;
- Did not address at least one of the specified review themes.

No restrictions were placed on the year of publication in order to capture historical trends and evolving policy and treatment strategies for MDR-TB in Central Asia.

### 4.2. Search Strategy

Standard search strategies utilizing five electronic databases (PubMed, Web of Science, Scopus, Embase, and WHO Global Tuberculosis Database and ClinicalTrials.gov) in a search for the relevant literature about drug-resistant TB in Central Asia were employed. The keywords used for database selection were identified using the Thesaurus dictionary terms and MeSH (Medical Subject Headings) terms.

The keywords used for all databases were as follows: Drug-resistant TB OR Drug-resistant tuberculosis OR Drug-resistant TB OR Multidrug-resistant TB OR MDR-TB OR Extensively drug-resistant TB OR XDR-TB AND Central Asia OR Central Asian region OR Central Asian countries OR Tajikistan OR Uzbekistan OR Kyrgyzstan OR Kazakhstan OR Turkmenistan OR Post-Soviet Central Asia AND Infection control prevention, OR Disease control measures OR Disease prevention strategies OR Infection prevention and control OR Hygiene practices OR Disease transmission prevention OR AND Infection control protocols OR Preventive healthcare measure OR Outbreak control OR Disease surveillance OR Epidemiological control OR Barrier precautions AND Diagnostic test OR Laboratory test OR Diagnostic procedure OR Diagnostic evaluation Diagnostic assay OR Diagnostic screening OR Diagnostic tool OR Diagnostic technique OR Diagnostic method OR Test procedure OR Diagnostic imaging OR Rapid diagnostic test OR Serological test OR Blood tests OR Pathological examination OR Histological analysis OR Microbiological culture AND Treatment management OR Therapeutic management OR Treatment administration OR Treatment adherence OR Treatment regimens OR Treatment approaches OR Disease management OR Clinical management OR Care management OR Patient management.

### 4.3. Study Selection

The screening was conducted in three phases:

1. Title and abstract screening: Two independent reviewers screened 12,584 titles and 8115 abstracts.
2. Full-text review: Selected articles underwent full-text evaluation based on eligibility criteria.
3. Final inclusion: 29 articles met the criteria.

Disagreements during the screening process were resolved through discussion and consensus. The inter-rater agreement was strong (Cohen’s kappa = 0.81).

### 4.4. Software and Equipment

Information Microsoft Excel 2019 (Microsoft Corporation, Redmond, WA, USA) was employed for data extraction and synthesis. No laboratory equipment or diagnostic devices were used, as this study is based on secondary analysis of published literature.

### 4.5. Quality Assessment and Synthesis

Methodological quality was assessed using the Kmet et al. [38] checklist, scoring studies from 0 (poor) to 2 (strong) across 14 criteria. All included studies received moderate-to-high quality scores. Due to methodological heterogeneity, no meta-analysis was conducted. Instead, a narrative synthesis was employed. The findings were organized into four themes aligned with the study objectives. Each theme was discussed in the context of regional health policies and challenges related to TB control.

## 5. Limitations

This review is subject to several methodological and contextual limitations that may influence the interpretation and generalizability of its findings. The scope of this review varies significantly in terms of research design, aims, population, inclusion criteria, and findings, affecting its generalizability. Although this review adhered to the PRISMA 2020 guidelines to enhance transparency and methodological rigor (Table S1), the absence of a specified timeline may have limited the inclusion of more recent research advancements. Next, the lack of a defined timeline may have prevented new research advancements. This review follows an integrative narrative synthesis approach rather than a systematic review due to the heterogeneity of available studies, variability in study designs, and lack of standardized reporting on MDR-TB interventions in Central Asia. While an integrative review offers broad thematic insights, it does not provide the quantitative rigor of a systematic review with meta-analysis. Future research should aim to conduct systematic reviews that incorporate meta-analytical methods, with a particular focus on longitudinal surveillance, real-time genomic data integration, and larger-scale epidemiological studies. Nevertheless, this review consolidates existing evidence, identifies research gaps, and provides critical insights into MDR-TB control in the region.

## 6. Conclusions

This review demonstrates that the Beijing genotype of MDR-TB is the predominant strain driving MDR-TB transmission in Central Asia, contributing significantly to regional resistance patterns. Key barriers to effective control include diagnostic delays, limited access to molecular tools, suboptimal treatment adherence, and the high cost of second-line therapies. Socioeconomic challenges, such as poor healthcare infrastructure and disease-related stigma, further complicate response efforts.

To address these gaps, national health authorities must expand access to molecular diagnostics, ensure the consistent availability of all-oral treatment regimens, and strengthen financial support mechanisms. Regional collaboration on genomic surveillance is also crucial for monitoring resistance trends and informing public health interventions. The findings highlight an urgent need to standardize surveillance systems, enhance healthcare worker training, and integrate TB services with broader social support structures to improve adherence.

Future research should prioritize early detection strategies, real-time monitoring of treatment efficacy, and in-depth investigation into the behavioral and structural determinants of adherence. Cross-border data sharing and a harmonized regional strategy will be pivotal in improving MDR-TB control and reducing the long-term public health burden across Central Asia.

## Figures and Tables

**Table 2:** Challenges, diagnostic tools, and treatment approaches for MDR-TB in Central Asia.

Country	Challenges and Barriers	Molecular Diagnostic Tools	Strategies for Infection Control and Treatment Approaches
Kazakhstan [11,14,20,25,29,31]	Rural underuse; cartridge shortages; training gaps High out-of-pocket costs (avg. USD 900); 92% report income loss; limited psychosocial support [29,31,33]	GeneXpert, LPA, WGS	Implementation of N95 masks, UVGI, HEPA filtration, and negative-pressure isolation rooms in major hospitals. Adoption of WHO-recommended all-oral MDR-TB regimens. Integration of DST and GeneXpert MTB/RIF for early detection [2,11,12,19,24,29,31,33].
Tajikistan [16,19,22]	Stockouts; poor maintenance; uneven rural coverage Delayed diagnosis from informal provider use; weak public–private linkage; stigma; diagnostic infrastructure gaps	GeneXpert	Hospital-based protocols involving isolation practices and ventilation improvements. Integration of TB-HIV care with ART. Progressive scale-up of oral treatment regimens per WHO guidelines [13,16,19,24,29,30,31].
Kyrgyzstan [10,22,28]	Limited sequencing capacity; ongoing phenotypic DST reliance. Continued use of conventional diagnostics; limited lab and HR capacity; inadequate staffing; disrupted supply chains	GeneXpert, LPA	GeneXpert introduction in central facilities. Airborne infection prevention via improved infrastructure and staff training. DOTS continuation with tailored second-line treatment [8,19,22,28].
Uzbekistan [6,9,13,23,30,31] [9,10,31]	Widespread reliance on phenotypic DST in remote regions Limited access to TB centers in rural areas; 24% SLDR-TB among RR-TB patients; reliance on phenotypic DST Stigma; treatment misconceptions; low health literacy	GeneXpert, LPA, limited WGS	Expansion of molecular diagnostics (GeneXpert, WGS), facility-based infection control (ventilation, PPE), and DOTS strategy reinforcement. Use of all-oral treatment with second-line agents like bedaquiline and linezolid [8,10,11,12,19,31].
Turkmenistan [6,7]	Diagnostic capacity likely limited; Beijing strains reported only	Unclear; likely phenotypic DST No recent data published; gaps remain undocumented	Basic infection control strategies reported. Beijing strain monitoring ongoing. Limited but increasing alignment with WHO recommendations on diagnostics and treatment regimens [7,19].

## Data Availability

Data sharing is not applicable.

## Supplementary Materials

The following supporting information can be downloaded at https://www.mdpi.com/article/10.3390/antibiotics14070673/s1, Table S1. PRISMA checklist. Figure S1. CIA map of Central Asia (1995). Reference [39] is cited in the Supplementary Materials.
