# Supplementary material for: Multidrug-Resistant Tuberculosis in Central Asia and Predominant Beijing Lineage, Challenges in Diagnosis, Treatment Barriers, and Infection Control Strategies: An Integrative Review
Source: Antibiotics (Basel). 2025 Jul 2;14(7):673. doi: 10.3390/antibiotics14070673 (PMC12291989; doi:10.3390/antibiotics14070673)
Supplement: Supplementary file 1 [file antibiotics-14-00673-s001.zip › SM Figure Supplemenntary file.pdf]

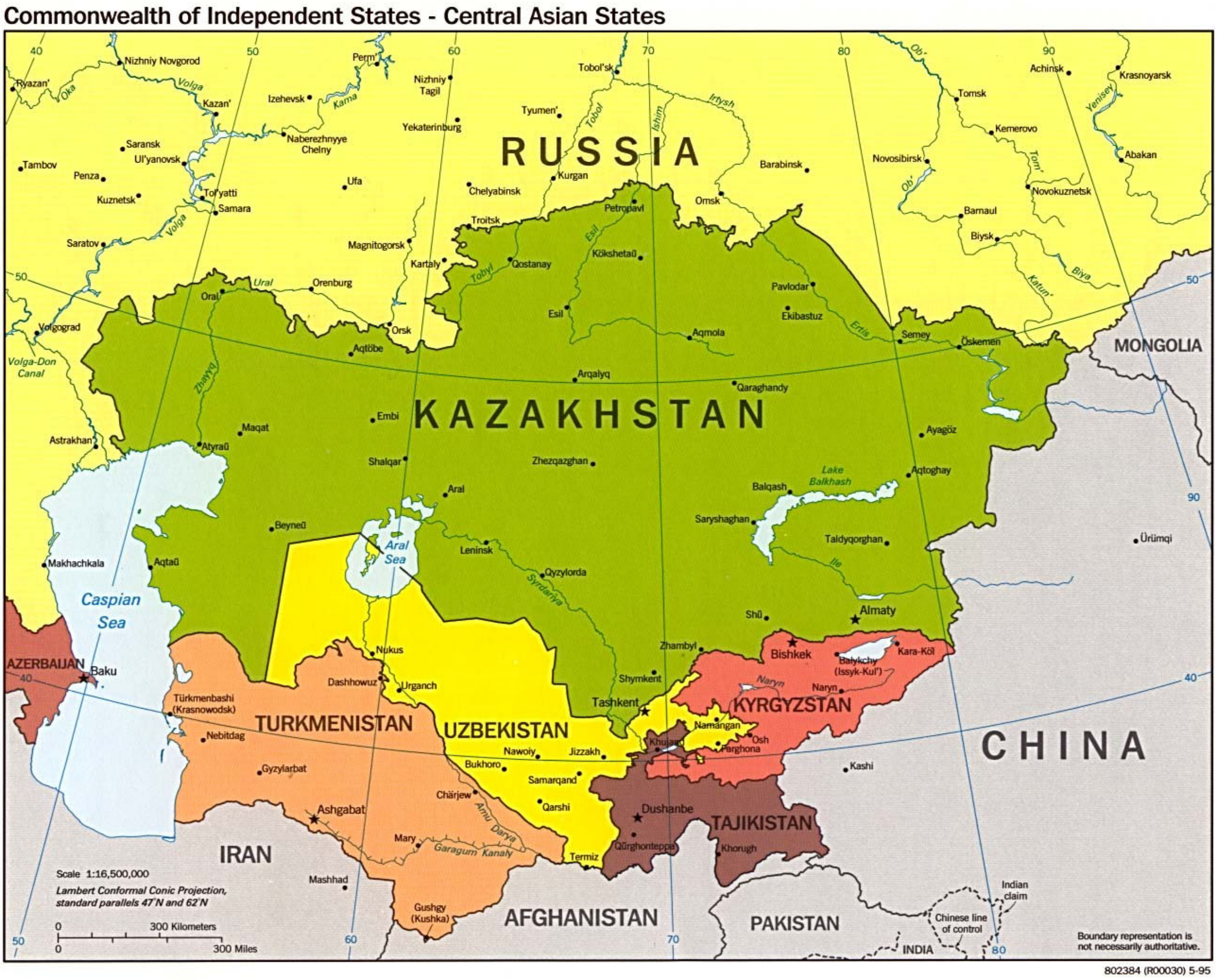

Figure S1. CIA map of Central Asia (1995), showing the geopolitical boundaries relevant to the MDR-TB burden. Source: Central Intelligence Agency, Perry-Castañeda Library Map Collection, Wikimedia Commons [Public Domain] [39]. Kazakhstan—Beijing genotype [11,14,16, 17]; Beijing Central Asian/Russian Cluster 94-32 genotype and endemic cluster KAZ-1 [4,14]. Tajikistan—Beijing genotype [20]; Beijing Cluster 100-32 and 94-32 [20]. Kyrgyzstan—Beijing genotype strains [19,20]. Beijing Cluster 94-32 [20]. Turkmenistan—Beijing genotype strains [7]. Uzbekistan—Beijing genotype strains [7,15,23,20,24,25]; Beijing Cluster 94-32 [20].
